# Outbreak of hepatitis A genotype IB in Australia associated with imported frozen pomegranate arils

**DOI:** 10.1017/S0950268818003515

**Published:** 2019-01-25

**Authors:** N. Franklin, H. Camphor, R. Wright, R. Stafford, K. Glasgow, V. Sheppeard

**Affiliations:** 1 NSW Ministry of Health, Sydney, Australia; 2 National Centre for Epidemiology and Population Health, Research School of Population Health, The Australian National University, Canberra, Australia; 3 Australian Government Department of Health, Canberra, Australia; 4 Queensland Health, Brisbane, Australia

**Keywords:** Disease outbreaks, foodborne infections, hepatitis A

## Abstract

Locally acquired hepatitis A infection is re-emerging in Australia owing to person-to-person outbreaks among men who have sex with men and imported frozen produce. This paper describes a multi-state foodborne outbreak in the first half of 2018. Enhanced human epidemiological investigation including a case–control study, as well as microbial surveillance and trace-back investigations concluded that the outbreak was caused by consumption of imported frozen pomegranate arils. A total of 30 cases of hepatitis A infection, genotype IB with identical sequences met the outbreak case definition, including 27 primary cases and three secondary cases. Twenty-five (83%) of the cases were hospitalised for their illness and there was one death. Imported frozen pomegranate arils from Egypt were strongly implicated as the source of infection through case interviews (19 of 26 primary cases) as well as from a case–control study (adjusted odds ratio 43.4, 95% confidence interval 4.2–448.8, *P* = 0.002). Hepatitis A virus (HAV) was subsequently detected by polymerase chain reaction in two food samples of the frozen pomegranate aril product. This outbreak was detected and responded to promptly owing to routine genetic characterisation of HAVs from all hepatitis A infections in Australia as part of a national hepatitis A enhanced surveillance project. This is now the third outbreak of hepatitis A in Australia from imported frozen fruits. A re-assessment of the risk of these types of imported foods is strongly recommended.

## Background

Hepatitis A virus (HAV) is primarily spread via the faecal-oral route, either by ingestion of contaminated food and water or direct contact with an infected person. The disease is characterised by fever, malaise, anorexia, nausea, abdominal pain and jaundice though some persons may have mild or no symptoms. Asymptomatic infection is particularly common in children under 5 years of age [1].

The incidence of HAV infection has markedly declined in Australia since the 1990s when the notification rate of HAV was consistently above 10 cases per 100 000 population, but since 2010 has averaged less than one case per 100 000 population [2]. Currently the most common source of HAV acquisition in Australia is in people who have travelled to high prevalence countries and among men who have sex with men (MSM). In Australia the HAV vaccine is recommended for people who are at higher risk for infection and since 2005 has been offered to indigenous children ⩽2 years in four Australian states [3].

Widespread foodborne hepatitis A outbreaks have occurred in Australia, the largest being in 1997 associated with contaminated locally produced oysters [4], followed by an outbreak in 2009 associated with imported sun-dried tomatoes [5]. More recently in 2015 and 2017 imported frozen berries were found to be the source of infection for two multi-state outbreaks in Australia [6, 7]. In addition, there has been a rise in locally acquired HAV among MSM in several Australian states and territories since 2017 related to the ongoing outbreak of HAV genotype 1A among this high risk group in Europe [8, 9].

In March 2018 a multi-state investigation was launched following the identification of locally acquired HAV cases in multiple Australian states. These cases had not travelled outside of Australia and reported no other high risk behaviour for HAV. Routine genetic sequencing of the virus identified a unique strain of HAV genotype IB that had not been seen in Australia previously. Initial case interviews using hypothesis generating questionnaires revealed high levels of consumption of frozen pre-packed fruits, with 100% of early cases having consumed the same imported frozen pomegranate aril product. This paper describes the investigation into this outbreak and the evidence confirming the source.

## Methods

### Case finding

Hepatitis A infection is a nationally notifiable disease under legislation in Australia and laboratories and medical practitioners must report confirmed cases to public health authorities. All cases are interviewed by trained public health officers using a national standardised questionnaire.

In Australia, a confirmed case of HAV is defined as a person with either laboratory definitive evidence (detection of HAV by nucleic acid testing) or laboratory suggestive evidence (detection of HAV-specific immunoglobulin M in the absence of recent vaccination) and clinical evidence or laboratory suggestive evidence and epidemiological evidence [10].

### Outbreak case definition

For the outbreak investigation, cases were classified as confirmed or probable.

A confirmed case was defined as a person who met the national hepatitis A surveillance case definition, genotyped as HAV IB with the characteristic outbreak genetic sequence, and with date of symptom onset (or date of testing if onset date not available) from 1 January 2018 and who must have spent some of their exposure period (15–50 days prior to onset of illness) in Australia.

A probable case was defined as a person who met the national hepatitis A surveillance case definition, with genotype IB but genetic sequence not yet compared (or met the probable national hepatitis A surveillance case definition [10] and had an epidemiological link to a confirmed outbreak case), and onset from 1 January 2018, and who must have spent some of their acquisition period (15–50 days prior to onset of illness) in Australia.

### Epidemiological investigation

A national standardised questionnaire is used for all HAV cases in Australia that focuses on food consumption and high risk activity for the acquisition of HAV. Following initial interviews this questionnaire was enhanced for further notified cases to include more questions on salad items and frozen fruit consumption. Initial cases were re-interviewed.

In addition, a prospective, frequency matched case–control study was conducted, in order to generate additional analytic evidence to support a causal association between consumption of the suspected source product and the outbreak of locally acquired HAV. This study was conducted under the Public Health Act for each jurisdiction so ethical approval was not required.

The confirmed case definition for the case–control study was consistent with the outbreak investigation case definition, except that cases were excluded if they were non-English speaking or unable to provide coherent answers to interview questions, unable to provide date of onset of illness or jaundice, or had close contact with a person known or suspected to have hepatitis A during the case's exposure period. One case was excluded due to not being able to provide a date of onset and two cases were excluded that were secondary cases. Cases notified between 13 April 2018 and 8 June 2018 were enrolled and interviewed using a standardised questionnaire containing questions on eligibility, consent and demographics and structured questions on a limited range of shellfish, dried fruits and fresh and frozen fruits. A probable case definition was also defined for the study, but was not required as all enrolled cases were confirmed.

Controls were recruited from jurisdictional notifiable disease databases, being either previously notified cases of salmonellosis, campylobacteriosis or cryptosporidiosis where necessary. It was attempted to frequency match controls to cases by age group (0–14, 15–39, ⩾40 years), in a 2:1 ratio. During this period, unrelated but concurrent disease outbreaks in multiple jurisdictions constrained public health resource capacity to continually recruit eligible controls; therefore a consensus decision was reached to close the case–control study once sufficient study power had been reached to demonstrate a statistically significant association between consumption of the implicated food source and HAV infection. To minimise selection bias, controls were recruited from the same or a neighbouring local government area as cases in order to account for potential variability in retail supply distribution of the suspected food source, and hence potential for exposure. To ensure the control was likely to be well and eating normally during the exposure period of the corresponding hepatitis A case, the specimen collection date for controls was required to be within the 2 weeks prior to the onset date of the corresponding hepatitis A case. Controls were questioned on potential exposures during the 5 week period prior to their onset of illness. Control exclusion criteria included: past infection with HAV; previous vaccination for HAV or receiving normal human immunoglobulin in the 2 months before date of diarrhoea onset; being an enteric pathogen case included in a separate outbreak investigation; not being contactable by telephone; non-English speaking or unable to provide coherent answers to interview questions; having travelled overseas in the 2 months prior to their date of diarrhoea onset; having lived in a country with high HAV endemicity for at least 1 year of the first 5 years of life; or having close contact with a known or suspected HAV case in the 2 months prior to their date of diarrhoea onset. All participants gave verbal consent to participate and were interviewed by telephone.

Questionnaire responses were entered into a centralised database, then exported and analysed using Stata™, version 13 [11]. Case and control demographic details were compared using Pearson *χ*^2^ and Wilcoxon rank sum test for gender and age respectively. Unconditional univariable analysis was used to calculate odds ratios (OR) and 95% confidence intervals (CIs) for the association between illness and foods consumed using Fisher's exact test, with *P* values <0.05 considered statistically significant. Where there was zero occurrence of an outcome for exposure to a variable in cases or controls, exact logistic regression was used to generate ORs and *P* values. Multivariable analysis employed stepwise logistic regression, with hepatitis A illness as the dependent variable, and food exposures with a *P* value <0.1 on univariable analysis included as independent variables, and excluded from the multivariable models in a reverse step-wise fashion. To account for frequency matching by age group, age was included in all multivariable models.

### Laboratory investigation

Serum samples for serologically diagnosed cases of HAV are routinely sent for further laboratory analysis. Samples were tested for HAV RNA by reverse transcription-polymerase chain reaction (RT-PCR) and if positive, HAV was sequenced employing the HAV network (HAVNET) protocol [12]. This procedure uses a nested RT-PCR to amplify a fragment of approximately 500 nucleotides spanning the HAV VP1/2A junction region which can be directly sequenced. After being analysed, the sequence data were uploaded to the National Centre for Biotechnology Information (NCBI) BLAST (Basic Local Alignment Search Tool) to determine the HAV genotype. Phylogenetic analysis compares isolate sequences against other sequences in the global HAVNET database.

### Food investigation

Opened and unopened packets of the implicated product belonging to the outbreak cases were collected for HAV testing as were unopened packets collected from retail stores and the supplier. The same product from a subsequent import batch that had not been repackaged or put out to sale was also sampled at the implicated factory. Other leftover frozen fruit products from cases freezers were also sampled opportunistically when they were volunteered by cases. Food samples were tested for HAV at the National Measurement Institute.

The testing method was based on ISO/TS Specification 15216-1, with Viral RNA Extraction by a Zymo Research ZR Viral RNA kit and HAV RNA sequence detection by a Genesig HAV Real-Time PCR kit. The sequence selected is proprietary but HAV specific. Controls included: positives (HAV sequence and internal spikes), negatives (template and extraction blanks) and replicates to monitor extraction and assay performance.

Controls included: positives (extraction blank spike, matrix spike, standard curve); negatives (extraction blank, non-template controls); internal control for all samples to ensure reverse transcription for the reverse transcription real time quantities PCR assay. Any food samples found positive for HAV were then forwarded to the laboratory that processed the human clinical samples to attempt to recover the HAV genotype and sequence by the method mentioned above.

The supply chain of the implicated product was traced back as far as possible by the Australian Federal Department of Agriculture. Local health authorities inspected the Australian food processing facility and assessed their operating procedures.

## Results

### Description of the outbreak

**Fig. 1.**
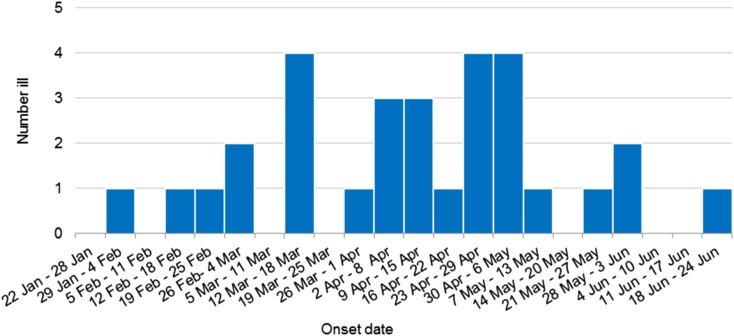
Weekly epidemic curve of confirmed outbreak cases by onset date (*n* = 30).

Thirty confirmed outbreak cases of HAV infection, genotype IB with identical sequences were identified during this investigation. Outbreak cases were reported from all but one Australian jurisdiction including New South Wales (15 cases), Victoria (six cases), Western Australia (three cases), Northern Territory (two cases), South Australia (two cases), Queensland (one case) and Australian Capital Territory (one case). Cases included eighteen females and twelve males, age range 4–74 years (median 30.5 years), with 25 of the 30 cases (83%) hospitalised for their illness. One death was reported, but the cause of death is yet to be determined. Three cases were secondary infections, epidemiologically linked to an earlier confirmed case and thought to have been acquired by sexual contact. The outbreak occurred over a 123 day period, onsets ranged from 31 January 2018 to 18 June 2018 (Fig. 1).

### Food consumption investigation

Table 1 describes the risk factors of cases. Food consumption was available for 26 primary cases, excluding the case that died. A high proportion of cases (23/26, 88%) reported purchasing groceries from one specific supermarket chain (supermarket A). Twenty-two of 26 (85%) primary cases reported consuming at least one type of frozen fruit. The median number of different frozen fruit products consumed per case was three (range 1–8). Consumption of frozen pomegranate arils was the most frequently reported food (18/26, 69%). Of these, 18/18 (100%) reported purchasing frozen pomegranates arils at supermarket A. This supermarket was the exclusive stockist of the product. Of the remaining eight cases: one case consumed pomegranate arils in a salad prepared at a cafe, which on investigation was revealed to be the implicated frozen pomegranate aril product. One case consumed salad containing pomegranate arils from several cafes but follow-up was not possible to find out what brand of arils were used in the salads. The final six cases could not recall eating pomegranate arils. Additionally the case that died had an unopened packet of the implicated product in her freezer.

**Table 1.** Risk factors among outbreak cases

Risk factor	Number exposed	Per cent exposed confirmed cases
All outbreak cases (*n* = 30)		
Contact with HAV case in previous 15–50 days	3/30	10
Travel outside Australia	1/30	3
Primary cases excluding the deceased case (*n* = 26)		
Food consumption		
Groceries from supermarket A	23/26	88
Groceries from supermarket B	15/26	58
Fresh berries – strawberry	12/26	46
Fresh berries – blueberry	12/26	46
Bagged lettuce – mixed leaf	15/26	58
Dried fruit – any	13/26	50
Shellfish – any	5/26	19
Tomatoes sun/semi-dried	4/26	15
Frozen mixed berries	8/26	31
Frozen raspberries	11/26	42
Frozen strawberries	10/26	38
Frozen blueberries	11/26	42
Frozen blackberries	3/26	12
Frozen cherries	5/26	19
Frozen mango	8/26	31
Frozen smoothie mix	8/26	31
Frozen coconut	2/26	8
Frozen pineapple	5/26	19
Frozen banana	3/26	12
Frozen acai puree	3/26	12
** Frozen pomegranate arils**	**18/26**	**69**
Cases who purchased frozen pomegranate arils (*n* = 18)		
** Frozen pomegranate arils purchased at supermarket A**	**18/18**	**100**

**Fig. 2.**
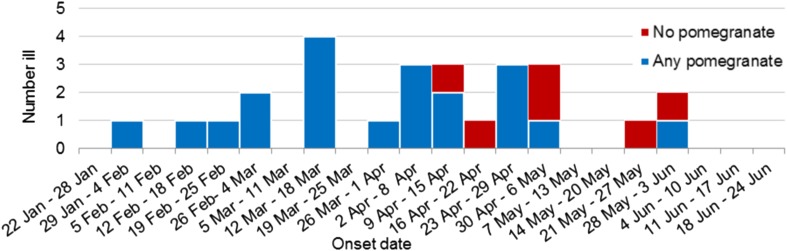
Weekly epidemic curve of primary outbreak cases with food exposure information by onset date and pomegranate aril consumption (*n* = 26).

Figure 2 shows the recall of any pomegranate aril consumption by primary cases over time. All cases (10/10) in the first half of the outbreak reported consumption of pomegranate arils whereas only 10/16 (62.5%) could recall eating any pomegranate arils in the last half of the outbreak.

### Case–control study

Thirteen cases (*n* = 13) and 21 controls (*n* = 21) were enrolled into the study, which ran for eight weeks, from 13 April 2018 to 8 June 2018.

There was no statistically significant difference in gender (*P* = 0.36); or age (*P* = 0.24) between cases and controls.

The results of the univariable analysis found that consumption of ‘frozen pomegranate arils’ had the strongest association and was significantly associated with illness (OR 45.0, 95% CI 3.8–2065.4, *P* < 0.001); as was consumption of ‘frozen strawberries’ (OR 20.6, 95% CI 2.6–∞, *P* = 0.001) and ‘frozen raspberries’ (OR 17.1, 95% CI 1.5–826.4, *P* = 0.007). No other items consumed were significantly associated with illness (Table 2).

**Table 2.** Univariate analysis of selected food exposures

Food item consumed	Cases		Controls		Crude OR	95% CI	*P* value
	*n*	%	*n*	%			
Shellfish							
Oysters^a^	0	0	2	9.52	0.7	0.0–8.7	0.513
Mussels	1	7.7	1	4.8	1.7	0.02–137.3	1
Other shellfish	2	15.4	3	14.3	0.8	0.07–10.6	1
Frozen fruits							
Frozen mixed berries	4	30.8	5	23.8	1.4	0.2–8.6	0.704
Frozen strawberries^a^	6	46.2	0	0	20.6	2.6–∞	0.001*
Frozen blueberries	6	46.2	3	14.3	5.1	0.8–38.9	0.057
Frozen raspberries	6	46.2	1	4.8	17.1	1.5–826.4	0.007*
Frozen pitted cherries	2	15.4	1	4.8	3.6	0.2–225.0	0.544
Frozen mango	4	30.8	3	14.3	3.0	0.4–24.7	0.377
Frozen pineapple chunks	4	30.8	2	9.5	4.2	0.5–52.5	0.173
Frozen banana chunks	1	7.69	2	9.52	0.8	0.01–16.9	1
Frozen acai puree^a^	1	7.69	0	0	1.6	0.9–∞	0.382
Frozen pomegranate arils	9	69.2	1	4.8	45.0	3.8–2065.4	<0.001*
Other frozen fruit^a^	2	15.4	0	0	4.5	0.92–∞	0.0478*
Other food items							
Other pomegranates^a^	0	0	0	1	0.8	0.5–0.9	0.015
Semi-dried tomatoes	3	23.1	2	9.5	2.7	0.3–36.4	0.364

a Exact logistic regression.

* Statistically significant at *α* = 0.05.

In the multivariable analysis models the only variables that remained in the final model were ‘frozen pomegranate arils’ and ‘age’ (OR 43.4, 95% CI 4.2–448.8, *P* = 0.002).

### Environmental investigation, product trace back and control measures

The implicated product was 180 g bags of frozen pomegranate arils. This product was stocked exclusively by supermarket A and distributed to all states and territories. Food Standards Australia and New Zealand (FSANZ) co-ordinated a national consumer level recall of the product on 7 April 2018. The product originated from a manufacturer in Egypt, repacked in Australia (transferred into branded packaging) and placed on the Australian market from October 2017 until the recall on 7 April 2018. All but one primary case in this outbreak had illness within one incubation period of the product recall. The one primary case that had an onset of 55 days after the recall was unaware of the recall and reported consumption of the product up until their onset date.

The investigation of the Australian food processor that repackaged the product in Australia found they were operating with all appropriate hygiene and food safety control processes, concluding that the local food processor had no process deficits to suggest it was the cause of the contamination, and that contamination most likely occurred before the product arrived in Australia.

Thirteen different packets of the implicated product were sampled by health authorities as were other frozen fruit packets surrendered by cases, including frozen banana [2], strawberries [1], coconut [1] and acai puree [1]. HAV was only detected in one opened packet of the pomegranate product and one unopened packet of a frozen banana product collected from the same case's house. An investigation suggested that the positive banana result was likely due to cross-contamination of specimens in the laboratory. Further testing of unopened banana chunks packets from the factory did not detect any HAV.

Testing of the subsequent imported batch of the implicated product that had not been repackaged or released to market was conducted by the Australian wholesaler and one of the 10 pomegranate arils sub samples from this testing was detected with HAV.

All the PCR positive food samples that were referred for further testing did not have HAV detected at the referring laboratory and so were unable to be genotyped or sequenced. This was likely due to the difference in test methods and test sensitivities used between laboratories.

Documentation provided by the Australian importing company suggested that the Egyptian manufacturer of the suspected source product was the same manufacturer linked to a 2012 outbreak of HAV genotype IB associated with imported frozen pomegranate arils in Canada [13]. In response to this outbreak the Australian Department of Agriculture and Water Resources commenced a 100% inspection and testing schedule of future consignments from the Egyptian manufacturer of the pomegranate arils linked to outbreak.

## Discussion

Australia experienced a nationwide outbreak of HAV in the first half on 2018 affecting 30 people (27 primary cases and three secondary cases). Analysis of the HAV sequence of the 30 cases identified all cases were HAV genotype 1B with an identical genetic sequence that was unique on the global HAVNET database. Based on food consumption history, imported frozen pomegranate arils were identified to be likely source of the infection with 19 of the 26 primary cases that could be interviewed reporting consumption of a specific product imported from Egypt. Additional evidence was obtained from the case–control study that demonstrated that the implicated frozen pomegranate aril product had the strongest association with illness. The detection of HAV RNA in food samples provided further support for frozen pomegranate arils being the source of the outbreak.

An enhanced national surveillance of hepatitis A project began in Australia in July 2017 prior to the detection of this outbreak. This project aims to gain improved understanding of the risk factors and molecular epidemiology of HAV in Australia, and to detect clusters of locally acquired hepatitis A to enable rapid public health action. The project was proposed after the national foodborne HAV outbreak associated with the consumption of frozen mixed berries in 2015 [6], where HAV sequencing assisted in identifying outbreak cases. A similar surveillance project in the Netherlands allowed health authorities to identify food-associated infections that would not have been detected otherwise, this included cases that could be linked to international outbreaks. Additionally, these potentially foodborne clusters were able to be identified without an increase in cases [14]. Prior to 2017, locally acquired cases in Australia were very rare, so an increase in locally acquired cases such as in 2009 with the sundried-tomato outbreak [5] and 2015 in the frozen mixed berries outbreak [6] stood out as unusual and prompted public health investigation. In 2017–2018, several states in Australia saw an increase of locally acquired HAV (genotype 1A) due to person-to-person spread, among cases with either male to male sex or injecting drug use as a risk factor [8, 9]. Fifty-three per cent of HAV cases in NSW in 2017 were locally acquired, a 250% increase on the proportion locally acquired in the previous 5 years [7]. Considering the small number of cases in this outbreak spread over many months, against a background of increased locally acquired cases, without genetic sequencing information this outbreak may not have been detected in such a timely manner.

A suspect food vehicle for this outbreak was detected 70 days after the onset of the first case, after nine cases had been reported, five of which were confirmed as having the HAV 1B genetic sequence. At this stage of the investigation all nine cases had reported eating the same nationally distributed frozen pomegranate aril product that was imported from Egypt, where HAV genotype 1B is reported to be common [15]. No other food had a similar high frequency of consumption, the biological plausibility and a geographic distribution that matched the cases in this outbreak. This was deemed sufficient evidence to implicate the food product and trigger a recall. A case–control study was nevertheless undertaken to provide additional analytic evidence to support the association between consumption of the product and illness. This was considered to be a prudent step because obtaining confirmatory microbiological evidence of HAV in suspect foodstuff is difficult due to the variable distribution of the virus on the food and owing to the level of the virus in the food being below the level that can be detected; this has been the case during previous HAV outbreak investigations in Australia and overseas [5, 16].

It was interesting to note that all 10 (100%) of the cases that fell sick prior to the recall could recollect eating any pomegranate arils whereas only 10 of 16 (63%) could recollect eating any pomegranate arils after the recall. Typically a nationwide recall and the media surrounding such a recall would bias recollection towards the implicated product, but in this case that did not happen. Given the certainty that the latter cases had the same source of infection based on the 100% homology of this sequence type in the outbreak cases, it may be that later cases who reported not eating pomegranate arils either did so as an ingredient in a food dish and didn't realise it or were in fact secondary cases to unidentified primary cases. Spill-over of HAV from food-exposed people to the general population is an important consideration in such investigations. Much of the public health intervention in individual HAV cases is follow-up of contacts for vaccination, but undiagnosed cases represent an additional risk for spill-over. A recent investigation of spill-over from a MSM outbreak in the Netherlands [17] found that for every two HAV cases with MSM risk factors notified with the MSM strain, there was one notified who did not have MSM risk factors. If this occurs during a foodborne outbreak it would mean that as time goes on the epidemiological investigation becomes more difficult as the strength between the relationship of consumption and disease weakens. This highlights the importance of being able to identify related cases early, when their exposure risks are more likely to be limited to the source food.

Even without spill-over effects, identification of a specific food vehicle can be difficult in outbreaks due to poor recall by cases, especially considering the long incubation period of HAV. Although imported, minimally processed foods that may be consumed without cooking are often the focus of HAV foodborne investigations, investigators cannot discount an infected worker potentially contaminating a local ready-to-eat product or ingredient. Initially bagged lettuce and fresh local blueberries both had high consumption rates in this investigation, but the feasibility of these locally farmed products creating the temporal and spatial pattern of infection was investigated and found to be untenable. Many different frozen fruit items were mentioned by a number of cases which led to the revision of the national questionnaire that included all imported frozen fruits that were currently on sale at the implicated supermarket. It was not until cases were specifically asked about these individual products that many recalled consuming them. An additional difficulty was then found as cases more often than not reported consuming numerous frozen fruit products. This could present a problem for identification of the source in the analytical analysis and may also raise concerns about cross contamination at the packing plant. In this outbreak the presence of the genotype 1B helped direct lines of investigation of the source as this genotype had been associated with HAV outbreaks with foods imported from Egypt [13] and Turkey [5, 16] and at the time of the investigation the only frozen fruits on shelves at supermarket A imported from that region were frozen pomegranate arils from Egypt and frozen cherries from Turkey.

HAV was detected in three food samples. One pomegranate aril sample that had not been repackaged or left the factory, one pomegranate aril sample and one banana sample which were collected from a case's house. The genotype was not able to be determined from these food samples, which is often the case in HAV outbreaks [13, 16]. However, the combination of other supporting descriptive epidemiological evidence, analytical epidemiological evidence and spatio-temporal distribution of the product indicates that the frozen pomegranate aril product was the source of the outbreak without this genotyping information.

Imported frozen pomegranate arils have been associated with HAV outbreaks previously, in Canada from an imported Egyptian product in 2012 [13] and in the USA from an imported Turkish product in 2013 [16]. Although it was discovered that the same Egyptian manufacturer likely provided the pomegranate arils for this current Australian outbreak and the 2012 Canadian outbreak, a comparison of the HAV genotype IB sequences from the two outbreaks showed seven nucleotide differences from an overlapping 363 nucleotides (97.5% homology) of the HAV VP1-2A region, indicating that they were unlikely to be genetically related. As no farm or processing facility trace back in Egypt was possible, it is not feasible to identify how the product became contaminated before entering Australia. However, evidence that pomegranate arils from this region have been previously associated with HAV contamination and that HAV is endemic in this area [15, 18] supports the likelihood of some pre-import processes being the source of the contamination. It is therefore reasonable to infer that minimally processed, ready-to-eat foods such as frozen fruit products that come from this HAV endemic area are susceptible to HAV contamination and may be of greater risk to susceptible consumers.

In a population like Australia with low background immunity there remains potential for further foodborne outbreaks of hepatitis A infection. A large number of cases in this outbreak were hospitalised (83%) and one person died, demonstrating that relatively small outbreaks such as this have the potential for serious morbidity, mortality and health system costs. Growing international food trade and the popularity of lower cost convenience foods means that imported frozen fruits will remain a commonly consumed product.

The onus for preventing unsafe food from coming into Australia is placed on the importer. It is an offence to import food into Australia if the importer knows, or ought reasonably to have known, that it poses a risk to human health. Importers must ensure that supply chains for foods have effective control strategies in the form of good agricultural practices and good hygienic practices. After the 2015 frozen berry outbreak, the Australian Department of Agriculture and Water Resources implemented a programme of inspection and testing on imported berries. However, this only includes tests for *Escherichia coli* as an indicator of overall process hygiene and only to fresh and frozen berries. It is not reasonable to test for HAV at the border due to the difficulties in detecting HAV in food described above. Importantly, although being of equal risk to frozen imported berries owing to the minimal processing of the product prior to consumption, other imported frozen fruit products including pomegranate arils, are not included in this screening process.

There is an urgent need for food safety recommendations in Australia to be re-assessed in relation to non-berry frozen fruit products. In the absence of control of HAV in imported food, consumers must be made more aware of the risks involved in consuming imported minimally processed ready-to-eat foods, such as imported frozen pomegranate arils and other frozen fruit products. They also need to be made more aware of the steps required to prevent disease, such as being vaccinated for HAV and the cooking steps required to eliminate pathogens such as HAV in food.

## Conclusions

Routine genetic sequencing of HAV is a necessary tool to detect and investigate outbreaks in Australia where person-to-person locally acquired infections are re-emerging in certain populations. Imported frozen fruits have now been associated with three recent outbreaks of HAV in Australia, and given the difficulty in detecting HAV in food and the growing popularity of such convenient health foods, consumers need to be informed of the risks of these products if they are consuming them without further cooking. A re-assessment of the risk of these types of imported foods to a susceptible population is strongly recommended.
